# A Review of Cocoa Drying Technologies and the Effect on Bean Quality Parameters

**DOI:** 10.1155/2020/8830127

**Published:** 2020-12-03

**Authors:** Banboye Frederick Dzelagha, Ngwabie Martin Ngwa, Divine Nde Bup

**Affiliations:** ^1^Department of Nutrition, Food and Bioresource Technology, College of Technology, University of Bamenda, Cameroon; ^2^Department of Agricultural and Environmental Engineering, College of Technology, University of Bamenda, Cameroon

## Abstract

Considering drying as a key farm-based, quality determining unit operation in the cocoa processing chain, this paper reviews recent studies in the drying methods and quality parameters of cocoa beans. Open sun, solar, oven, microwave, and freeze drying methods have been investigated at various levels in the drying of cocoa beans with objectives to improve the drying properties and final quality of cocoa beans. While an open sun dryer employs natural passive mechanisms, the solar drying methods can employ a combination of passive and active mechanisms. The oven, microwave, and freeze drying methods are fully active requiring electrical energy inputs. To improve drying rates in the open sun method, dryer materials and location of drying trays are the parameters optimized since the drying temperature depends on solar intensity. For solar dryers, materials, angles of elevation, heaters, and fans are manipulated to optimize energy absorption and drying parameters. For the oven and microwave methods, drying air properties are directly controlled by electronic systems. Moisture content, mouldiness, bean colour, pH, titratable acidity, fat content, and acetic acid concentration are the most widely evaluated bean quality parameters.

## 1. Introduction

Cocoa (*Theobroma cacao*) beans constitute a global raw material for the chocolate industry, beverages, cosmetics, pharmaceuticals, and toiletry products [1]. Over fifty million people depend on cocoa for their livelihood with a global production capacity of 68% for Africa, 17% for Asia, and 15% by the Americas [2, 3]. In Africa, the largest cocoa-producing countries by volume are Ivory Coast (1900 million tonnes (MT)/ear), Ghana (850 MT), and Cameroon with 250 MT of global supply in the cocoa market [4]. This contributes significantly to the gross national incomes of these countries. The physics and chemistry of cocoa beans are very complex and change throughout its life span depending on the processing method and geographical origin [1]. As such, cocoa beans of commercial grade should conform to specified criteria among which are its moisture content, acidity, slatiness, polyphenol content, mouldiness, and mycotoxin production [5].

Cocoa bean quality depends on each of its production processes, from farming practices, region of origin, and transportation to industrial facility where transformation occurs. Fermentation and drying constitute key farm(er)-based unit operations with strong influences in the final quality of cocoa beans and subsequent products. Recent studies on the drying process and effects on quality point to three principal issues—method, temperature, and duration of drying [6]. Variations of these drying parameters impinge significant effects on moisture content, bean colour, pH, fatty acids, polyphenols, methylxanthines, proteins, and aromatic compounds that constitute outstanding quality parameters (Tables 1(a)–1(d)) [6, 7].

Although fermentation and drying have complementary influences on bean quality, a poor drying process of well fermented cocoa beans can result in beans of very poor quality since heat treatments affect bean quality parameters differently [8]. In an attempt to optimize the drying process and obtain optimal cocoa bean quality with minimal cost, several modifications in drying parameters have been carried out. This review focuses on the recent innovations in the cocoa drying process and effects on quality parameters. It starts with a brief description of the most popular drying methods used in the drying of cocoa beans, recent advancements in the drying technology, and effects on bean quality parameters and ends with a tabulated summary of the drying methods and quality parameters.

## 2. Cocoa Drying Methods

Any drying system which minimizes exposure of food to light (ultraviolet (UV)), oxidation, and heat will help conserve critical bioactive compounds required for high produce quality [9].

During drying, the water activity of food is reduced due to a reduction in its moisture content through the application of heat [10]. Cocoa beans are considered dry and suitable for marketing if the moisture content has been reduced to between 5 and 8% (wet base) [11]. As reviewed by Bala and Nipa [12] and Naseer et al. [9], five types of drying systems employed in the cocoa sector have been identified, namely, the open sun, solar, oven, microwave, and freeze drying. These are briefly reviewed below, as the basis on which various works have been carried out aimed at improving the drying potentials of the dryers and the quality of the dried cocoa beans.

### 2.1. Open Sun Drying

Sun drying is the oldest, cheapest, most popular, and freely available method that can be applied using the most rudimentary to highly sophisticated and scientific procedures, especially in the tropics and subtropics where solar radiation is abundant [12]. Over 60% of African and Asian cocoa is dried locally using the open sun [3], the main reason for the usually higher moisture content at the time of sale by the farmers. Among renewable energy resources, solar energy is considered indispensable in the future, as it is inexpensive, abundant, inexhaustible, environmental friendly, and nonpollutant [13].

Open sun drying is widely carried out by spreading the beans on raised wooden mats and plastic sheets or on concrete floors during sunshine (Table 1(a)). The beans are manually turned regularly, and someone needs to stay by to scare away the biotic contaminants and also to carry the beans to the shade in case of rain. Though freely available, this method is weather dependent, is labour intensive, takes longer (7-22 days), and exposes food to vermin and other environmental contaminants [14]. This increases the risk of giving poor quality beans compared to the more controlled drying methods, as longer drying periods expose the beans to mould growth [15]. Some poorly informed farmers dry their cocoa beans on a bare ground (exposing them to contamination by stones, soil, and surface organisms) and some on tarred road sides (exposing them to contamination by carcinogenic compounds) [3, 8]. Though the cheapest and most freely available to every farmer, this method is not practically feasible during periods of heavy rain and high humidity.

### 2.2. Solar Drying

According to Fagunwa et al. [16] and Sneha [17], solar dryers are devices that use solar energy to dry substances, especially food using solar energy. The dryer heats the air to a constant temperature, and the heat generated is used to dry the produce items in the drying chamber and also heats up the surroundings. The air used for drying is passed through a collector surface, gets heated, and is then used to dry the food item inside the dryer. While the produce is exposed to solar radiation and wind in open sun drying, it is contained in an enclosed space and protected from direct wind in solar dryers. Ventilation is enabled at a constant rate through defined air inlets and outlets, small solar ventilators, or temperature difference, either due to exposition or due to vertical height [18]. Although solar dryers are mostly used to dry cocoa beans at experimental and research levels, a few farmer corporative groups in Asia have used the greenhouse dryers to dry cocoa beans in a large scale [12].

As reviewed by Sivakumar and Rajesh [19] and Sneha [17], together with several workers, they have identified four types of solar dryers (Table 1(b)): the *direct solar dryer* (e.g., solar box dryer) is where the item to be dried is exposed directly to solar radiation through a transparent material that covers the structure. The heat generated from the solar energy is used to dry the produce and also heats up the surroundings. The *indirect solar dryer* (e.g., solar cabinet dryer) is where the solar radiation is absorbed and converted into heat by another surface (like a black top) usually called the collector. The air that will be used for drying is passed over this surface and gets heated, which is then used to dry the food item inside the dryer. The *mixed-mode dryers* (e.g., solar tunnel dryer) employ the use of the direct and indirect passive sources of heat energy to dry the produce, while the *hybrid solar dryers* (e.g., hybrid solar/biomass cabinet dryer) employ the passive and active sources of heat energy to dry the produce.

Where some form of intervention is applied to control the drying temperature, such as the use of electric fans to influence air flow, the dryers are described as active, in contrast to the passive modes where the drying temperature is fully dependent on natural weather. Although it is more expensive and complex to construct compared to direct solar dryers, some of its advantages include conservation of produce from external contaminants, less human labour, and availability in varied sizes. Based on the design of system components and mode of utilization of solar energy, three types of solar drying systems have been reviewed [19, 20].

The natural convection solar dryer (passive dryers) is a portable direct type which consists simply of a rectangular shaped with a transparent top and blackened interior surfaces. A clear polyethylene plastic is placed over the heat collector to allow solar radiation to heat the air. A black material is also placed under the chamber to absorb the heat and to keep out moisture from the ground. Another black polyethylene sheet could also be placed over the drying chamber to prevent bleaching. Ventilation holes may not be provided along the sides, but an opening in the front of the unit allows ambient air to enter the heating chamber, and another opening at the rear of the drying chamber allows moist air to escape from the unit by natural convection air movements. This dryer is unlikely to be suitable for drying the large quantities of coca produced by farmers, though it may give acceptable experimental results.

The forced convection dryer (active dryers) is a direct mode forced convection dryer which essentially consists of a blower to force the air through the product and a chamber and is covered with a transparent sheet. Indirect mode forced dryers essentially consist of an air heater, a drying chamber, and a blower/fan to duct the heated air to the drying chamber. The drying chamber may consist of several trays to contain thin layers of drying produce.

The third type, a hybrid solar dryer, is a modification of the two types above and includes the solar drying system with thermal storage such as the forced convection greenhouse solar dryer. The solar drying system with auxiliary unit is composed of an electric heating collector, a drying chamber, a fan, and an auxiliary heater. The hybrid with geothermal or waste waters employs a drying agent as a heating source but could use solar energy, geothermal or waste waters, a conventional source, or conventional and unconventional energy sources. Other modifications include the solar drying system with photovoltaic, solar drying system with heat pump [21].

A solar drying system with a chemical heat pump consists of four mean components—solar collector (evacuated tubes type), storage tank, chemical heat pump unit, and dryer chamber. A cylindrical tank could be selected as a storage tank. The chemical heat pump unit contains the reactor, evaporator, and condenser. The general working of chemical heat pump occurs in two stages: adsorption and desorption.

### 2.3. Oven Drying

A drying oven is a type of low-temperature convection or forced air oven used primarily in laboratory settings. Specimens, tools, and temperature-sensitive chemicals are placed inside a drying chamber to slowly and evenly remove moisture. An electric dehydrator is a self-contained appliance with a heat source, a fan for circulation, and multiple trays for drying many foods at one time. Better quality dehydrators also have thermostats and double-walled construction for more efficient energy usage [22]. As such, any researcher can dry any produce at any temperature for experimental purposes (Table 1(c)).

A local firewood oven dryer was constructed in Fako, Cameroon, with bricks and cement plastered and a tilting platform to support the suspended heating and combustion chamber above the ground [3]. To control the effect of smoke, the combustion chamber was separated from the drying chamber with 2 m heat/air conveying ducts (the kiln), in which dry wood was burnt to generate heat. The drying mat was perforated to facilitate the free circulation of air in the drying chamber. The rotation rates of the cocoa beans during the drying process and the rate of free exit of saturated air were the same as the rate of entry of hot/dry air into the drying chamber. Although this gave encouraging results (7% within 96 hours of continuous drying), the difficulty to control smoke and maintain the drying temperatures at 35-40°C and equality in the distribution of heat were some of the risks observed in the experimental process. Further studies are still expected on the dimensions of other cocoa bean quality parameters such as PAHs, acidity, mould content, and OTA content in Cameroon; over 40% of total cocoa productivity is dried using firewood ovens [3].

### 2.4. Microwave Drying

Microwave drying is a process that basically works in the same way as when we heat food in a microwave oven. The microwaves cease at the same moment as the drying machine or microwave oven is switched off. According to Feng et al. [23], microwave drying is based on a unique volumetric heating mode facilitated by electromagnetic radiation at 915 or 2,450 MHz. The responses of a lossy food product to dielectric heating result in rapid energy coupling into the moisture and lead to fast heating and drying. A significant reduction in drying time in microwave drying is often accompanied by an improvement in product quality, making it a promising food dehydration technology. The need for improvement in engineering design and process optimization for microwave drying has stimulated the development of computer simulation techniques to predict temperature and moisture history and distribution in the product to be dried. Despite the experimental successes that this method may achieve in the drying of cocoa beans, it is limited to very small-scale drying and practically unsuitable for unelectrified rural areas and large-scale production of cocoa (Table 1(d)).

Microwave drying relies on additional energy being supplied that is preferentially absorbed by the solvents in the process to enhance evaporation. Microwaves are a form of electromagnetic energy (300 MHz–300 GHz), generated by magnetrons under the combined force of perpendicular electric and magnetic fields. Microwave heating is a direct heating method. In the rapidly alternating electric field generated by microwaves, polar materials orient and reorient themselves according to the direction of the field. The rapid change in the field at 2450 MHz causes rapid molecular reorientation resulting in friction and heat. Different materials have different properties when exposed to microwaves, depending on the extent of energy absorption, which is characterized by the loss factor [24].

### 2.5. Freeze Drying

First used in 1949, freeze drying is a special form of drying that removes all moisture and tends to have less of an effect on a food's taste than does normal dehydration. In freeze drying, food is frozen and placed in a strong vacuum. The frozen water in the food turns to vapour (sublimation) [25, 26]. Although this method has been proven suitable in the experimental drying of cocoa beans, it seems practically impossible to be used in the large-scale drying of cocoa beans due to its high electrical and technical inputs.

## 3. Effect of Drying Method on Some Quality Parameters of Cocoa Beans

Cocoa has been savoured for over 3500 years for its rich flavour, ability to uplift mood, and capacity to increase energy. According to Ishaq and Jafri [27], Mandl [28], and HerbaZest [29], nutritional and health benefits of cocoa and its products include supporting brain health, a good source of antioxidants, regulating blood cholesterol level, and treatment for diabetes and bronchial asthma; reduce obesity; regulate cardiovascular health; treat constipation; prevent cancer; and support skin health. For cocoa beans to qualify for use by the chocolate and pharmaceutical companies, an analysis of the following quality parameters is required: moisture content (5.5-8% wet basis), pH (3.8-5.5), fat content (55-56%), shell content (15-17%), brown bean colour, free from moulds, high antioxidant activity, phenolic compounds, OTA, titratable acidity, acetic acid, PAH, theobromine, caffeine, dry bean recovery rate, foreign matter, bean count, sound of dry grain, ordour, and separation of husk [4, 7, 30]. Drying being a key quality determinant unit operation in the cocoa chain, variations of drying methods, temperature, and duration of drying have been extensively studied in relation to effects on quality attributes [6, 7].

Using the solar cabinet, a microwave oven, and open sun dryers, the drying characteristics of cocoa beans were evaluated. Although a high cut test score of 94.5% was obtained in all the dryers, free fatty acid content was the highest (9.42) in beans from the tray dryer while pH was higher than normal in the solar cabinet and microwave oven dryers (6.3 and 7.2) [31]. In decreasing rating of quality, cocoa beans from the tray dryer were the highest, followed by that from the solar cabinet, the open sun, and the microwave. Despite the significant reduction in drying time, the microwave dryer could be considered not suitable for drying cocoa beans based on bean quality output.

To evaluate the influence of drying methods on antioxidant activity and Ochratoxin A in cocoa beans, Deus et al. [1] dried properly fermented beans using four comparative solar dryers—one with stainless steel platform and plastic roof with UV protection, the second with a wooden platform and an artificial heat source (60°C), a traditional dryer in barge with wooden platform and drying by direct sun light, and a mixed dryer with stainless steel platform and mobile plastic roof without UV protection and exposure to sunlight. A reduction in antioxidant activity, i.e., phenolic compound content (catechin from 0.04 mg·g^−1^ to 0.02 mg·g^−1^) and methylxanthines (theobromine from 19.44 to 11.71 mg·g^−1^) was observed with only one sample showing contamination by OTA (7.1 *μ*g·kg^−1^). Despite the modifications in the drying methods, the traditional drying method showed the highest conservation of antioxidant activity, methylxanthines, and phenolic compound contents. These methods took a considerably long period of seven days to dry cocoa beans to the required 7% moisture content (wet basis) and, as such, may not be considered very suitable for large scale drying due to the increased risk of quality loss.

The performances and effects of open sun, oven, and mixed sun and oven drying methods on the chemical quality attributes of raw cocoa were evaluated [32]. The open sun and mixed dryers gave favourably lower free fatty acid (<0.70%) and free acidity contents. Cocoa beans from the oven and mixed dryers were more acidic (pH 3.8–5.2) compared to the more suitable acidity (pH 4.5-5.5) from the open sun dryer. Despite the acceptable bean quality, these results were not conclusive as drying time and temperature were not monitored as expected.

To compare the effects of open sun and oven drying (temperatures 35, 40, 45, 50, and 55°C) on the quality of fermented cocoa beans, the assessment of acetic acid level, pH, bean colour, free fatty acid, acid value, and grading was carried out [14]. Although sun-dried samples gave better results than the oven dried at all temperatures, the significant increase of free fatty acid and acetic acid levels with increase in temperature was directly proportional the corresponding decrease in pH, with optimal oven temperature being 45°C. Due to the required expertise and high operational cost, this oven method may not be recommended for the large-scale drying by rural farmers.

Using a solar greenhouse dryer (at temperature of 21–52°C), fermented cocoa beans were dried to 5.3% moisture content (wet basis) within seven days [33]. Although the results were not encouraging due to the long drying time and a low cut test score and brown fat content of 74% and 50%, respectively, the enclosed nature of the greenhouse eliminates the risk of mould and external bean contamination by rain and vermin. This could be advantageous to rural farmers if properly constructed and drying properties enhanced.

Using a heated batch drier at 55, 70, and 81°C under isothermal conditions, the drying kinetics of foreign cocoa species were investigated in Abia State, Nigeria [34]. Moisture content of batches was reduced to 5, 3, and 4% wet basis within 12, 6, and 4 hours of drying. In a similar investigation, a convective hot air dryer at 60, 70, and 80°C reduced the moisture contents of cocoa beans to 6.3, 5.7, and 3.6%, respectively, within eight hours [35]. Despite the significant reduction in drying time, drying cocoa beans at temperatures above 60°C is discouraged due to high retention of acetic acid and increase in bitterness [14, 31].

The influence of fermentation and drying materials on the Ochratoxin A (OTA) content in cocoa beans was evaluated using the open sun on rack tables, concrete floor, and black tarpaulins [36]. The OTA content increased from 0.275 ± 0.2 *μ*g/kg (during fermentation) to 0.569 ± 0.015 *μ*g/kg (during drying), but no significant relationships between the OTA level and the materials used in the fermentation and drying were established.

Using a direct solar dryer to investigate the effect of different loadings (20, 30, and 60 kg), the moisture content of cocoa beans was reduced to 7.5% in 5, 7, and 9 days of drying, respectively [37]. The pH of the dried beans was within range (5.1, 4.9, and 5.4), a reduction in titratable acidity (from 25.75 to 17.80, 18.57, and 13.30), and increase in mouldiness (light, moderately heavy to extremely heavy) in the 20, 30, and 60 kg loadings, respectively. Together with other quality assessments (bean colour, odour (vinegary, alcohol, faecal, rancid, cheesy, and sock), fermentation index, bitterness, and sourness), the 20 kg treatment produced better quality beans compared to other loadings and therefore is recommended for the direct solar dryer. Sacrificing quantity for quality disqualifies this drying method for use by large-scale farmers.

To investigate the effect of drying methods on the concentration of PAHs in cocoa butter, fermented cocoa beans were dried to a moisture content of 7.5% in the open sun, electric oven (80°C), and a combination of sun and oven drying [15]. Analytical results showed an increase in PAH concentrations during the drying process. Sun-dried beans had the least PAH concentration (0.23 to 0.5 ppb) and oven drying (the highest (0.25 to 1.18 ppb)). These increases were not statistically significant and thus not responsible for contamination of cocoa butter by PAHs. Since PAH concentrations were highest in dried bean shells than in the cotyledons, he asserted that PAH contamination of cocoa butter originates largely from smoke during drying since it migrates from the shell to the cotyledons during drying.

To investigate the effect of grain size and heat source on the drying profile of cocoa beans, Onwuka and Nwachukwu [38] cleaned and sorted fermented cocoa beans into four different grain size samples and dried in four different dryers (sun, oven, solar, and sweat box drying (400 W bulb)) to a moisture content of 13-14% (wet basis) within 19, 17, 9, and 6 hours, respectively. Sweat boxes with 400 and 300 watts were most effective in terms of time economy and could be used to dry cocoa during rains, but other quality parameters should be considered. Varying temperatures and humidity showed no significant differences in drying rates which were the same for all grain sizes. The thickness of layers rather than the bean size exhibited significant effects in the drying rates of cocoa beans. This method could be recommended for use by farmers if further studies are carried on large-scale drying.

Yeboah [39] and Manoj and Manivannan [40] independently developed MATLAB-based mathematical modeling and simulation to predict the air flow properties and equilibrium moisture content of greenhouse dryer for cocoa bean drying. Fermented cocoa beans were successfully dried from a moisture content of 50 to 7% (wet) in seven days. Although the beans were of grade one quality in terms of moisture content, the drying period was considered too long, and other quality parameters equally needed to be evaluated. Drying for up to seven days increases the chances of bean contamination by moulds and OTA.

To evaluate the efficiency of a polycarbonate tunnel-type solar dryer, by forced convection in the drying of cocoa beans, a 1000 kg capacity parabolic roof greenhouse dryer covered with polycarbonate sheets and concrete floor was constructed [41]. Solar cell-activated fans were integrated to homogenize internal temperature. 12.5 kg of fermented cocoa beans were successfully dried from a moisture content of 56.4 to 6.6% (wet basis) in twenty-four hours. A producer satisfaction test in terms of grain colour, ordour, separation of husk, mould appearance, and sound of grains gave a satisfaction index range of eight to ten on all criteria, indicating approval by cocoa producers. To ensure effective use by rural farmers, he recommended further evaluation of construction cost and payback period as a plausible perspective.

Using the open sun and a plastic roof greenhouse dryer constructed with local materials, a comparative study of the drying of fermented cocoa beans was carried out in Rural Colombia [20]. A reduction in moisture content from 58 to 7% (wet basis) was obtained in six and four days, respectively, indicating the efficiency of the greenhouse dryer. With the beans enclosed from external contaminants, rain, and low construction cost, the greenhouse dryer is more suitable for use by rural farmers than the traditional open air sun drying. Further studies on modifications to raise the drying temperature of the greenhouse dryer could cause a further reduction in drying time.

Using the fleece soil solarisation method to modify the conventional greenhouse dryer, Banboye et al. [42] investigated the drying kinetics of fermented cocoa beans. The significant temperature difference of 5.2°C of the modified greenhouse dryer above that of the conventional greenhouse dryer enabled the reduction of moisture content from 48.42 to 5.95% compared to 9.06% (wet basis) in the conventional greenhouse dryer after four days of drying. The reduction in drying time, the suitable bean colour, pH, and high cut test score of 97% brown beans could qualify this dryer for use by large-scale farmers, but a detail modelling, simulation, construction cost, and payback period still need further investigations.

Jewe et al. [43] investigated the potential improvements that the microwave-assisted roasting of nibs could have in terms of cocoa quality and energy consumption over convective roasting. Operating the microwave oven at 820, 615, 410 and 205 W, it was observed that the application of energy input resulted in more homogenous heating of cocoa beans with a suitable moisture content and bean colour. The total energy consumption was reduced by one-third compared to convective roasting. Although the microwave could be seen as conducive for drying and giving high-quality cocoa beans, it is expensive and fully dependent on electricity and requires expertise which is lacking in most cocoa farming communities.

In a comparative modelling of thin layer drying kinetics of cocoa beans using the open sun and microwave oven operated at 3200, 2800, and 2400 W by Verdier et al. [44], the moisture content was reduced from 53.52 to 5.5, 5.6, 5.6, and 5.7% (wet basis) in 35 hours and 8, 12, and 16 minutes, respectively. Similarly, Arsène et al. [45] used a domestic microwave at 700 W, 600 W, and 450 W power levels to characterize the moisture transport mechanism of cocoa beans during microwave pulse drying. The moisture content of fermented beans on rotating discs was reduced from 57.35 to 7.5% (wet basis) in 78, 100, and 180 minutes, respectively, indicating that mass transfer was more rapid within cocoa bean sample, at higher microwave power levels.

Further studies by Zamrun and Nyoman [46] employed a domestic microwave oven operated at 600, 300, and 150 W and sliced cocoa beans to a maximum of 3 mm thick with 10 mm diameter. The moisture content was reduced from 54 to 7% (wet basis) in 4.2, 5.8, and 7.5 minutes, respectively. This indicated that slicing cocoa beans before drying gave a significant reduction in drying time (to about half) and six to sixteen times less electrical energy (from 3200 W in 8 minutes to 600 W in 4.2 minutes and from 2400 W in 16 minutes to 150 W in 7.5 minutes). With the high electromagnetic radiation of 2,450 MHz used, it would be necessary for other quality parameters of the dried beans to be analyzed before the drying method could be conclusive for cocoa beans. Slicing the cocoa beans before drying limits the use of this method for large-scale drying. Some volatile components could equally escape during the slicing process leading to further loss in quality.

## 4. Conclusion

In the decreasing order of popularity, the solar, open sun, oven, microwave, and freeze dryers have been widely studied in the drying of cocoa beans, at various levels. The open sun dryer employs solely the natural and passive mechanisms and is weather dependent. The solar, including greenhouse dryers, can be adapted to function using either passive or active mechanisms or both. The oven and microwave methods are fully active and require consistent artificial energy inputs. To improve drying rate using the open sun method, the materials used and location of the dryer are the key parameters investigated since the drying temperature depends solely on natural solar intensity. For the solar dryers, various materials, angles of elevation of collectors, electric heaters, and fans are manipulated to optimize solar energy absorption and control drying temperatures. The oven and microwave methods use internal drying air properties that can be directly controlled by electrical energy inputs. Temperature and drying time are key parameters with direct effects on cocoa bean quality. In terms of suitability and possible usage for large scale drying by local farmers, the open sun and greenhouse dryers are the most preferred due to simplicity, low construction, and running costs. Moisture content, pH, titratable acidity, acetic acid, fat content, bean colour, mouldiness, phenolic compounds, OTA, and PAHs are the most common bean quality parameters evaluated in most studies.

## Figures and Tables

**Table 1:** Cocoa drying methods and frequently analyzed quality parameters.

S. No	Drying method(s)	Tem (°C)	Duration	MC (%)		pH	Fat (%)	Shell content	Other quality parameters analyzed	References
				BD	AD					
Set standards (reference values)		≤60	Varied	NA	5.5-8	3.8-5.5	55-56	15-17%	Bean colour, antioxidant activity, phenolic compounds, OTA, titratable acidity, acetic acid, PAH, mouldiness, theobromine, caffeine, dry bean recovery rate, foreign matter, bean count, sound of dry grain, ordour, separation of husk	[4, 7, 30]

(a) Open sun drying methods										
1	Direct sun dryer (wooden platform)	*>60*	7 d	52.2	*8.03*	NA	NA	NA	Antioxidant activity, phenolic compound content (catechin and methylxanthines), OTA	[1]
2	Open sun drying	NA	20 h	67	*8.6*	NA	NA	NA	Bean colour, dust, dirt, and other pollutants	[47]
3	Sun drying on raised bamboo mats	NA	8 d	53.16	5.5	6.7	NA	NA	Bean colour, titratable acidity, ash content	[48]
4	Open sun-drying with tempering	30.2 to 37.8 °C	60 h	NA	≤23	5.37–5.39	NA	NA	Bean colour, fat content, crude protein, carbohydrates	[49]
5	Open sun drier	NA	40 h	57	6.5	NA	NA	NA	Bean colour, flavour	[50]
6	Open sun drying	NA	NA	NA	NA	NA	NA	NA	PAHs = 6.22 *μ*g·kg^−1^	[51]
	Sun drying + wood smoke								PAHs = 114.92	
7	Open sun drying on black tarpaulin and bamboo mats.	NA	10 d	53.5	5.49	*5.9*	NA	NA	Polyphenol content, mould infection, bean colour, and OTA	[8]
8	Open sun drying	NA	NA	50	7	5.18	NA	NA	Total amino acid content, total sugar reduction, ordour	[52]
	Combined sun and tunnel dryer		18 h			*5.58*				
9	Full sun drying	NA	2-5 d		7.5	NA	NA	NA	PAH	[15]
10	Sun drying on rack tables, concrete floor, black tarpaulins	NA	NA	NA	7.5	NA	NA	NA	OTA (0.569 ± 0.015 mg/kg)	[36]
11	Open sun drying	NA	NA	53.7	6.97	*6.9*	NA	NA	FFA, acetic acid	[14]
12	Open sun drying	NA	NA	53.7	7-8	5	NA	NA	Volatile acidity, free acidity	[32]
	Mixed sun & oven drying					4.5				
13	Open sun drying	NA	30 h	49.9	7-8	5.1	NA	NA	Bean colour, titratable acidity, acetic acid, & free fatty acid	[31]
14	Direct sun dryer (wooden platform)	*>60*	7 d	52.2	*8.03*	NA	NA	NA	Antioxidant activity, phenolic compound content (catechin and methylxanthines), OTA	[1]
	Mixed dryer (plastic roof without UV protection and exposure to sun light)	>60		49.4	7.5					

(b) Solar drying methods										
1	Solar greenhouse dryer	30.9	7 d		5.7	5.2	*50*	*12.1*	Dry bean recovery rate, foreign matter, bean count, bean colour, brown fat	[4]
2	Greenhouse dryer (plastic roof with UV protection)	60	7 d	52.2	*8.1*	NA	NA	NA	Antioxidant activity, phenolic compound content (catechin and methylxanthines), OTA	[1]
		*>60*			6.9					
	Dryer with wooden platform	60			7.76					
	Mixed dryer (plastic roof without UV protection and exposure to sun light)	>60		49.4	7.5					
3	Cocoa house replica dryer	31-35	4.8d	50.9	6-8	5.46–4.97	NA	NA	Bean colour, water activity, cocoa flavour, total acidity, bitterness, astringency	[53]
	Greenhouse dryer		6.3d							
4	Heated batch cocoa bean dryer	55	6 h	79.6	6	NA	NA	NA	NA	[34]
		*70*	5 h							
		*81*	4 h							
5	Forced-air, artificial intermittent dryer	35, 40, 45, 50, & 55	NA	NA	NA	4.7-5.2	NA	NA	Free fatty acid, acetic acid levels	[54]
6	Solar tunnel heat pump dryer	29.6-48.8	20 h	67	8.6	NA	NA	NA	Bean colour, dust, dirt, and other pollutants	[47]
7	Solar drying	NA	8D	53.6	4.8	6.9	NA	NA	Bean colour, titratable acidity, ash content	[48]
8	Tunnel dryer	50,55,60, *65, 70, 75,80, & 85*	NA	NA	7-4.9	6.08-5.7	*12.02-10.12*	NA	Protein (%), fibre, ash, & CHO	[55]
9	Solar dryer for intermittent drying + forced convection	31-54	72 h	53.4	3.6	5.18	NA	NA	Acid value, bean colour, bitterness, mouldiness, free fatty acid level, moisture reabsorption rate	[16]
	Solar dryer for intermittent drying +natural convection		NA	NA	NA	6.35				
10	Solar dryer with adsorbent	40-54	41 h	NA	NA	NA	NA	NA	Weight stability	[56]
	Solar dryer with absorbent		30 h							
11	Oven drying with curing of 24 h after 3 hours of continuous drying	60	52 h	NA	13–14	5.37–5.39	NA	NA	Bean colour, fat content, crude protein, carbohydrates	[49]
12	Mobile solar dryer	NA	32 h	57	6.5	NA	NA	NA	Bean colour, flavour	[50]
13	Rotary dryer equipped with an electric heater and infrared lamps	50	9.25 h	56	7	NA	NA	NA	Polyphenols, caffeine, theobromine, epicatechin and catechin content	[6]
		55	8.5 h							
		60	7.1 h							
14	Mechanical (tunnel) dryer	60	NA	50	7	5.46	NA	NA	Total amino acid content, total sugar reduction, ordour	[51]
15	Indirect heavy load solar dryer	40-70	7 d	55	7.5	NA	NA	NA	Titratable acidity, bean colour, ordour	[57]
16	Integrated solar dryer with adsorbent desiccant	NA	30 h	56.4	6.3	NA	NA	NA	Mouldiness, bean colour, sound of dry grain, ordour, separation of husk	[41]
	Polycarbonate tunnel solar dryer (equipped with electric fans)		24 h							
	Integrated solar dryer with absorbent desiccant		19 h							
17	Solar drying	NA	9 h	58	13-14	NA	NA	NA	NA	[38]
	Sweat box drying (100 W bulb)		9 h							
	Sweat box drying (200 W bulb)		8 h							
	Sweat box drying (300 W bulb)		7 h							
	Sweat box drying (400 W bulb)		6 h							
18	Direct solar dryer at three levels of loading	20 kg	5 d	54	7.5	5.1	NA	NA	Mouldiness, titratable acidity	[37]
		30 kg	7 d			4.9				
		60 kg	9 d			5.4				
19	Tray dryer	NA	8 h	49.9	6.4	5.2	NA	NA	Bean colour, titratable acidity, acetic acid, & free fatty acid	[31]
	Solar cabinet dryer,		16 h	49.4	7.6	6.3				
20	Greenhouse dryer (plastic roof with UV protection)	60	7 d	52.2	*8.1*	NA	NA	NA	Antioxidant activity, phenolic compound content (catechin and methylxanthines), OTA	[1]
		*>60*			6.9					
	Dryer with wooden platform	60			7.76					

(c) Oven drying method										
1	Mechanical oven	40	4.3d	50.9	6-8	5.46-4.97	NA	NA	Bean colour, water activity, cocoa flavour, total acidity, bitterness, astringency	[53]
2	Humidity controlled dryer chamber (laboratory oven)	60	12, 24, 32, & 40 h	NA	NA	NA	NA	NA	Polyphenol contentment, astringency, flavour, bitterness	[58]
3	Wood smoke	NA	NA	NA	NA	NA	NA	NA	PAHs = 142.05	[51]
4	Oven drying	*60-80*	12-40 h	55	7.5	NA	NA	NA	Polyphenol content	[59]
	Adsorption drying	60	24 h							
	Vacuum drying	60	24 h							
5	Full electric oven	80	2-5 d		7.5	NA	NA	NA	PAH	[15]
6	Oven drying	35	39 h	53.7	6.85	6.7	NA	NA	FFA, acetic acid	[14]
		40			6.75	6.1				
		45	33 h		6.70	5.8				
		50			6.62	4.9				
		55	18 h		5.3	4.5				
7	Oven drying	60	NA	53.7	7-8	4.5	NA	NA	Volatile acidity, free acidity	[32]

(d) Microwave and freeze drying methods										
1	Microwave drying	3200 W	8 mins	53.5	5.60	NA	NA	NA	NA	[44]
		2800 W	12 mins		5.63					
		2400 W	16 mins		5.77					
2	Freeze drying (fresh beans blanched at 90°C for 5 minutes)	-30°C	24 h	55	7.5	NA	NA	NA	Polyphenol content	[59]
		-50°C	4 h							
	Vacuum drying	60	24 h							
3	Domestic microwave oven	600 W	250 sec	56.4	7	NA	NA	NA	NA	[46]
		300 W	350 sec							
		150 W	450 sec		6.6					
4	Microwave oven	NA	0.4 h	49.9	7.1	*7.2*	NA	NA	Bean colour, titratable acidity, acetic acid, & free fatty acid	[31]

Key: d = days; h = hours; mins = minutes; sec = seconds; EE = electrical energy; W = watts; NA = not available; MC = moisture content; BD = before drying; AD = after drying. The italic data show contents that fall below or above the set standards.
